# Supplementary material for: Fine-tuning of auxin homeostasis governs the transition from floral stem cell maintenance to gynoecium formation
Source: Nat Commun. 2017 Oct 24;8:1125. doi: 10.1038/s41467-017-01252-6 (PMC5654772; doi:10.1038/s41467-017-01252-6)
Supplement: Supplementary file 1 — Supplementary Information [file 41467_2017_1252_MOESM1_ESM.pdf]

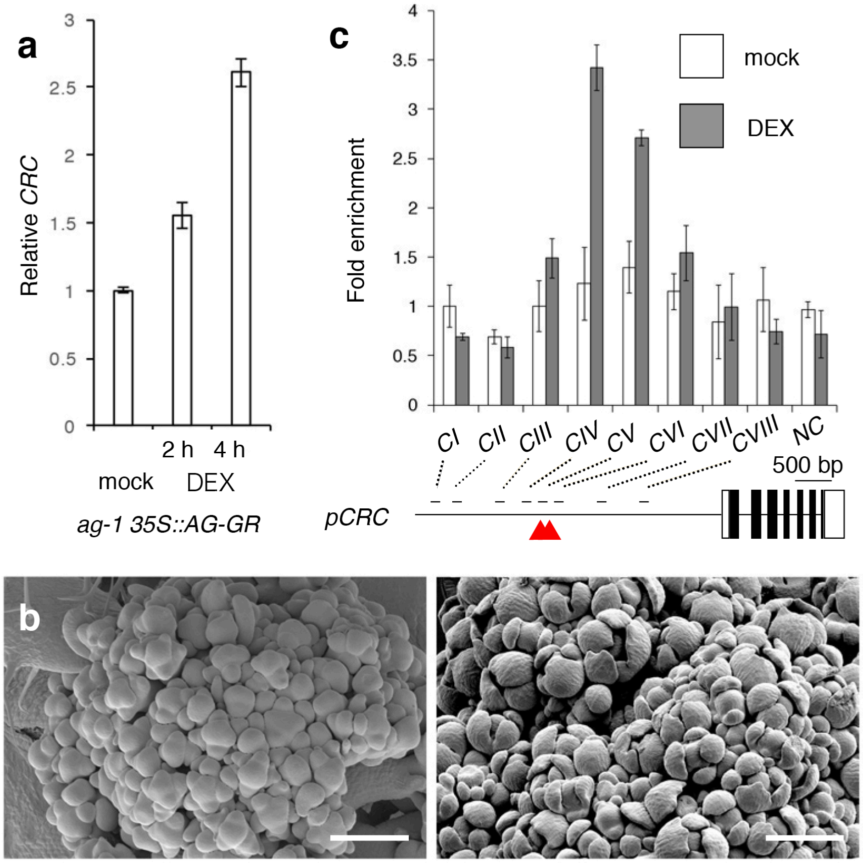

Supplementary Figure 1. Confirmation of AG protein association at the *CRC* promoter in stage 6 floral buds.

a, *CRC* mRNA levels in mock- and DEX-treated *ag-1 35S::AG-GR* floral buds. Activation of AG-GR by dexamethasone led to rapid induction of *CRC* expression. **b**, Synchronization of floral stage. Mock-treated *ap1-1 cal-1 35S::AP1-GR* (left). DEX-treated *ap1-1 cal-1 35S::AP1-GR* (right). The photo was taken 4 days after treatment, which is the proper time for ChIP analysis. The use of *ap1-1 cal-1 35S::AP1-GR* plants allowed us to enrich floral buds at developmental stage 6. **c**, Anti-AG ChIP in *ap1-1 cal-1 35S::AP1-GR* after synchronization of flower stages. In stage 6 floral buds-enriched tissues, AG strongly associated with the CIV region of the *CRC* promoter where previously identified CArG boxes are located. PCR fragments and *CRC* locus architecture are shown below the ChIP data. Red triangles indicate conserved CArG boxes. The *Mutator-like* transposase family gene (*At4g03870*) was included as a negative control (NC). Bar = 100 µm in **b**.

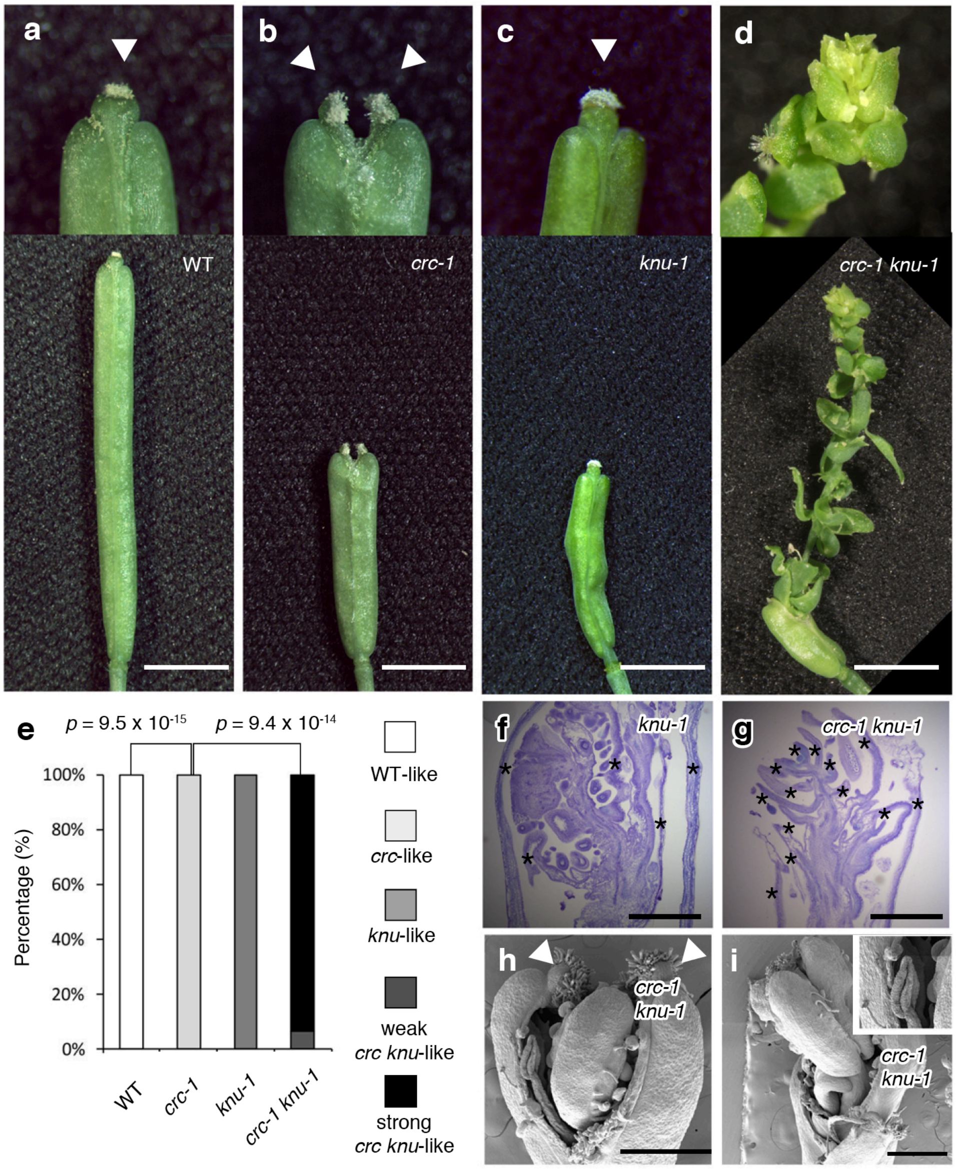

Supplementary Figure 2. ***CRC*** **and** ***KNU*****, downstream genes of AG, control floral meristem termination.**

a–**d** Morphology of wild-type (**a**), *crc-1* (**b**), *knu-1* (**c**), and *crc-1 knu-1* (**d**) fruits. Top: Close-up views of fruit tips. Bottom: Shapes of whole fruits. Arrowheads indicate stigma structures. **e**, Quantification of mutant phenotypes. *p*-values were calculated by Chi-square test (n = 30). **f**, **g**, Longitudinal sections of *knu-1* (**f**) and *crc-1 knu-1* (**g**) fruits. Asterisks indicate carpels. **h**, **i**, Scanning electron micrographs of *crc-1 knu-1* fruits with reiterations of floral organs. Weak *crc-1 knu-1* fruits phenotype (**h**). Strong *crc-1 knu-1* fruits phenotype (**i**). Stamens are shown in the inset. Bars = 1 cm in **a**–**d** bottom; 500 μm in **f**, **g**; and 1 mm in **h** and **i**.

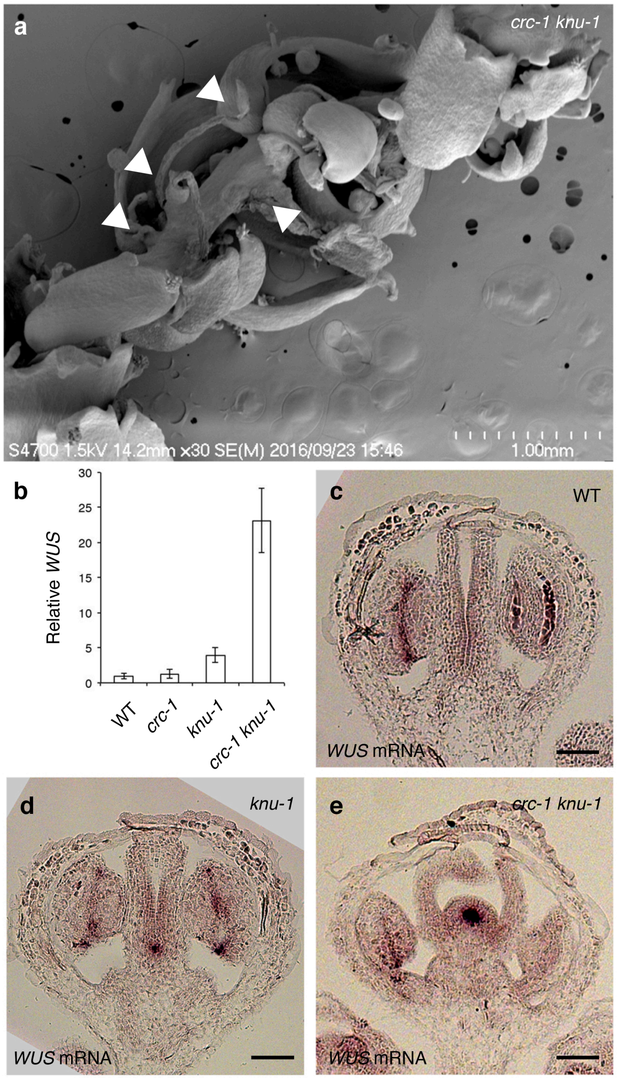

Supplementary Figure 3. Evaluation of *WUS* expression by RT-PCR and *in situ* hybridization.

a, Scanning electron micrograph of *crc-1 knu-1* fruits with reiterations of floral organs. Arrowheads indicate organ formation at different places on the internode. **b**, mRNA abundance of the stem cell marker *WUS* in wild-type, *crc-1*, *knu-1*, and *crc-1 knu-1* flowers. The mean and standard error of the mean of *WUS* expression normalized to that of *EIF4* (*At3g13290*) are shown. **c-e**, *WUS* mRNA in longitudinal sections of wild-type (**c**), *knu-1* (**d**), and *crc-1 knu-1* (**e**) flowers at developmental stage 9. *WUS* expression persists in *knu-1* and *crc knu-1* flowers. Bar = 50 μm in **c-e**.

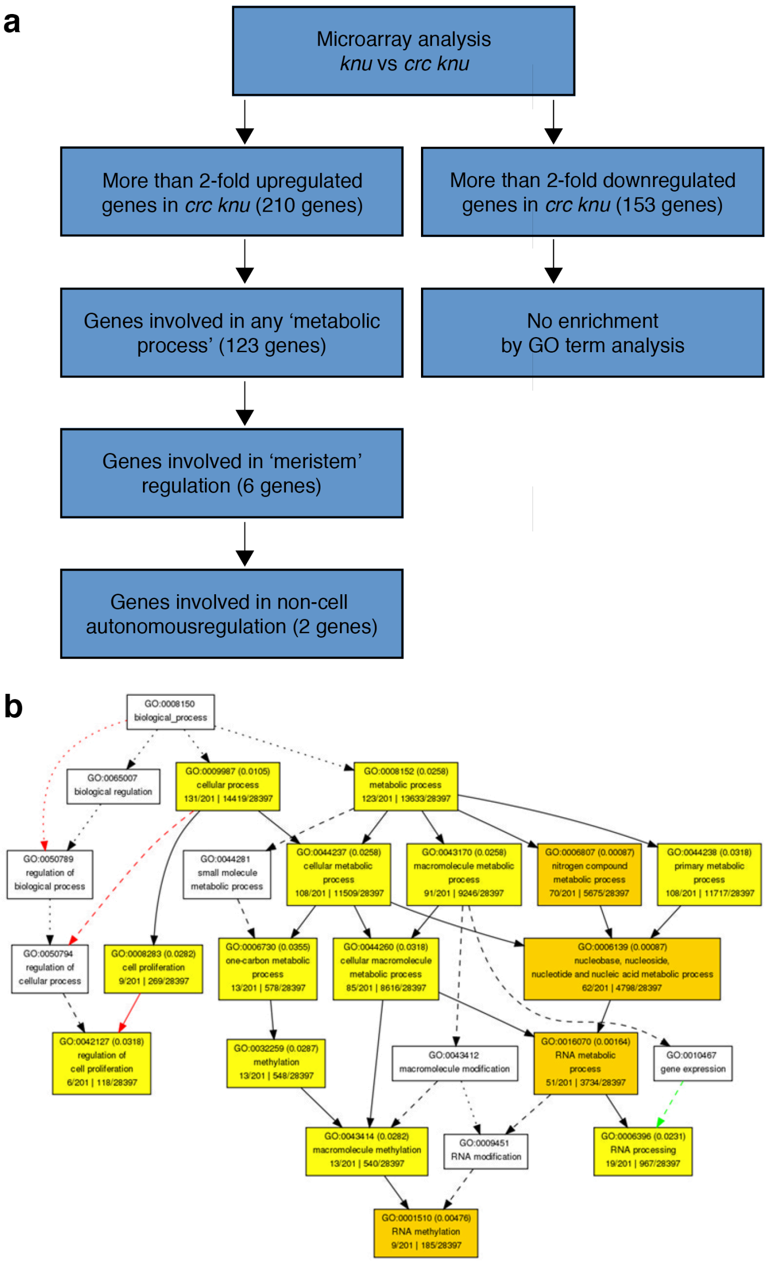

Supplementary Figure 4. Identification of CRC targets by microarray and GO term analyses.

a, Flowchart of CRC target identification pipeline. **b**, Hierarchical tree graph of over-represented GO terms in the biological process category.

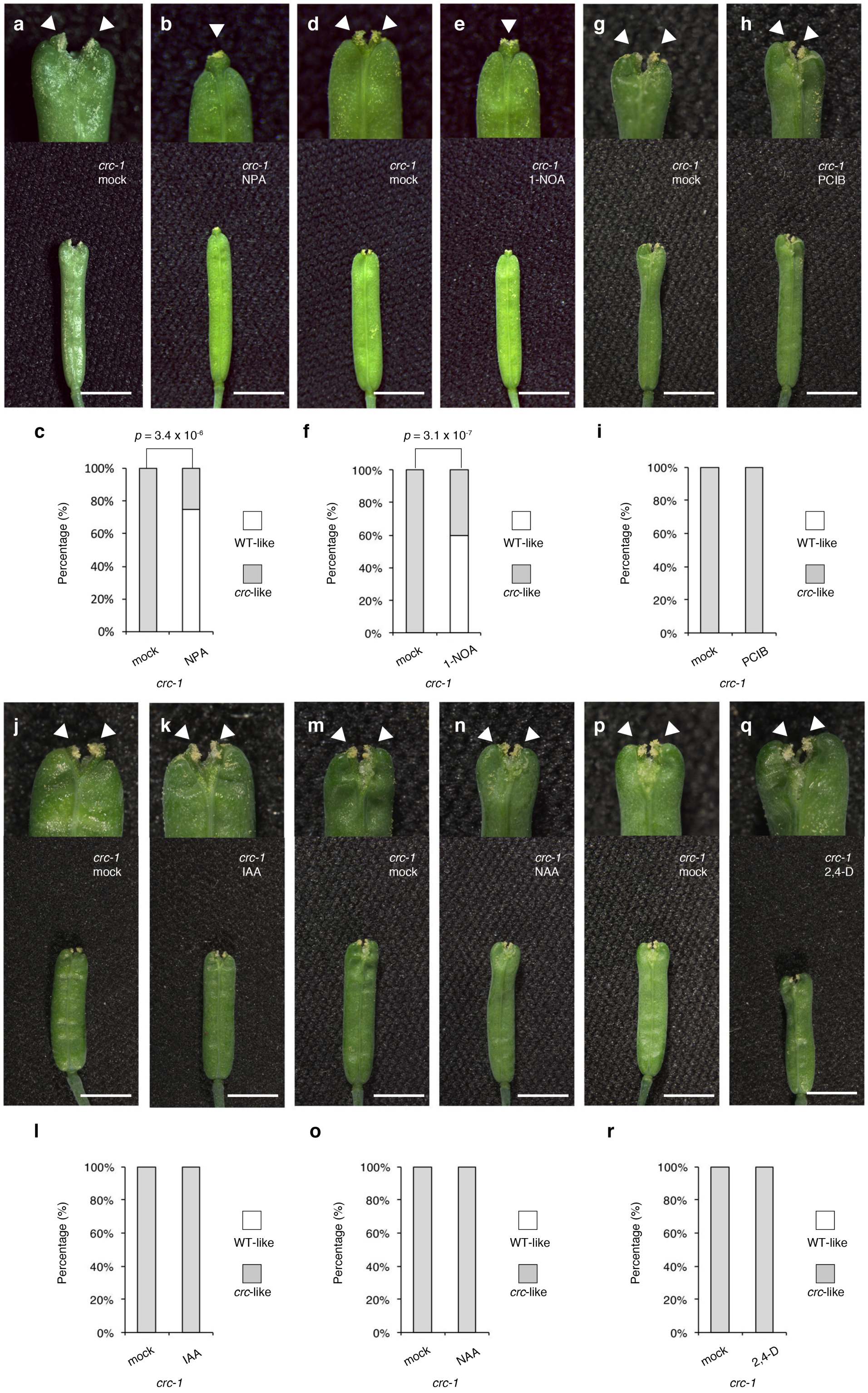

Supplementary Figure 5. ***crc*** **mutant phenotypes after auxin and inhibitor treatments.**

a-c, Morphology of mock-treated *crc-1* (**a**) and NPA-treated *crc-1* (**b**) fruits. Above: Close-up views of fruit tips. Below: Shapes of whole fruits. Quantification of mutant phenotypes after NPA treatment (**c**). **d-f,** Morphology of mock-treated *crc-1* (**d**) and 1-NOA-treated *crc-1* (**e**) fruits. Quantification of mutant phenotypes after 1-NOA treatment (**f**). **g-i,** Morphology of mock-treated *crc-1* (**g**) and PCIB-treated *crc-1* (**h**) fruits. Quantification of mutant phenotypes after PCIB treatment (**i**). **j–l,** Morphology of mock-treated *crc-1* (**j**) and IAA-treated *crc-1* (**k**) fruits. Quantification of mutant phenotypes after IAA treatment (**l**). **m-o,** Morphology of mock-treated *crc-1* (**m**) and NAA-treated *crc-1* (**n**) fruits. Quantification of mutant phenotypes after NAA treatment (**o**). **p-r,** Morphology of mock-treated *crc-1* (**p**) and 2,4-D-treated *crc-1* (**q**) fruits. Quantification of mutant phenotypes after 2,4-D treatment (**r**). Arrowheads indicate stigma structures. Two stigma-like structures were observed in the *crc-1* mutant due to carpel separation. *p*-values were calculated by a Chi-square test (n = 30). Bars = 1 cm in **a**, **b, d, e, g, h, j, k, m, n, p, q** bottom.

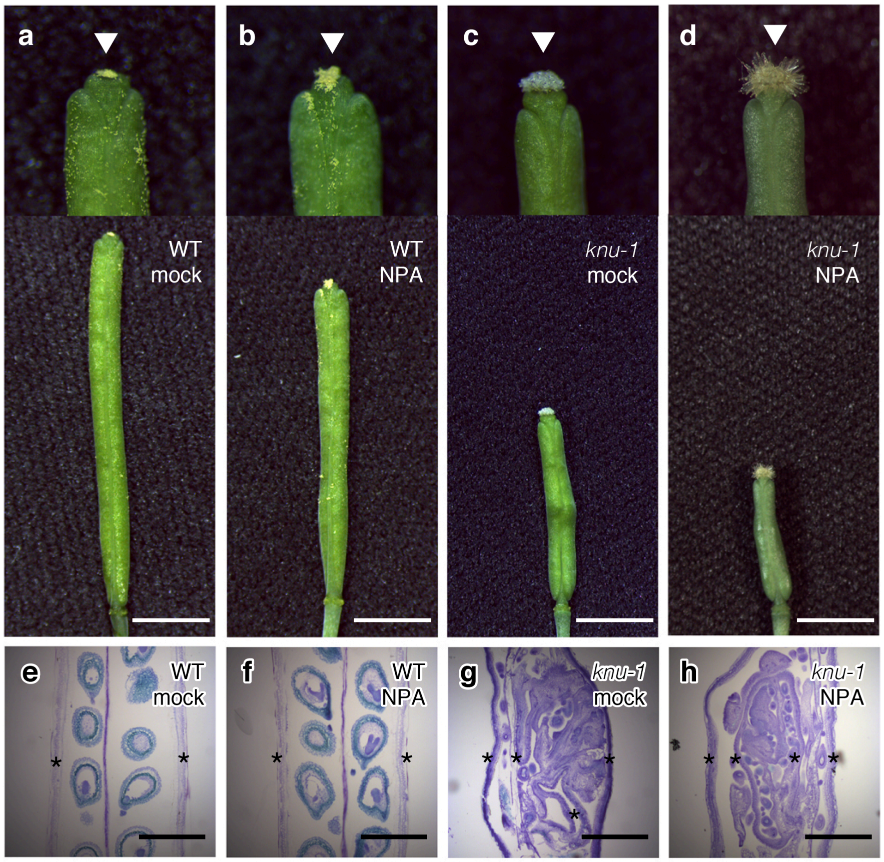

Supplementary Figure 6. Wild-type and *knu-1* double mutant phenotypes after NPA treatment.

a–**d** Morphology of mock-treated wild-type (**a**), NPA-treated wild-type (**b**), mock-treated *knu-1* (**c**), and NPA-treated *knu-1* (**d**) fruits. Top: Close-up views of fruit tips. Bottom: Shapes of whole fruits. Arrowheads indicate stigma structures. **e**-**h**, Longitudinal sections of mock-treated wild-type (**e**), NPA-treated wild-type (**f**), mock-treated *knu-1* (**g**), and NPA-treated *knu-1* (**h**) fruits. No morphological changes except for slightly shorter lengths were observed in wild-type or *knu-1* fruits treated with NPA. Asterisks indicate carpels. Bars = 1 cm in **a**–**d** bottom; and 500 µm in **e**–**h**.

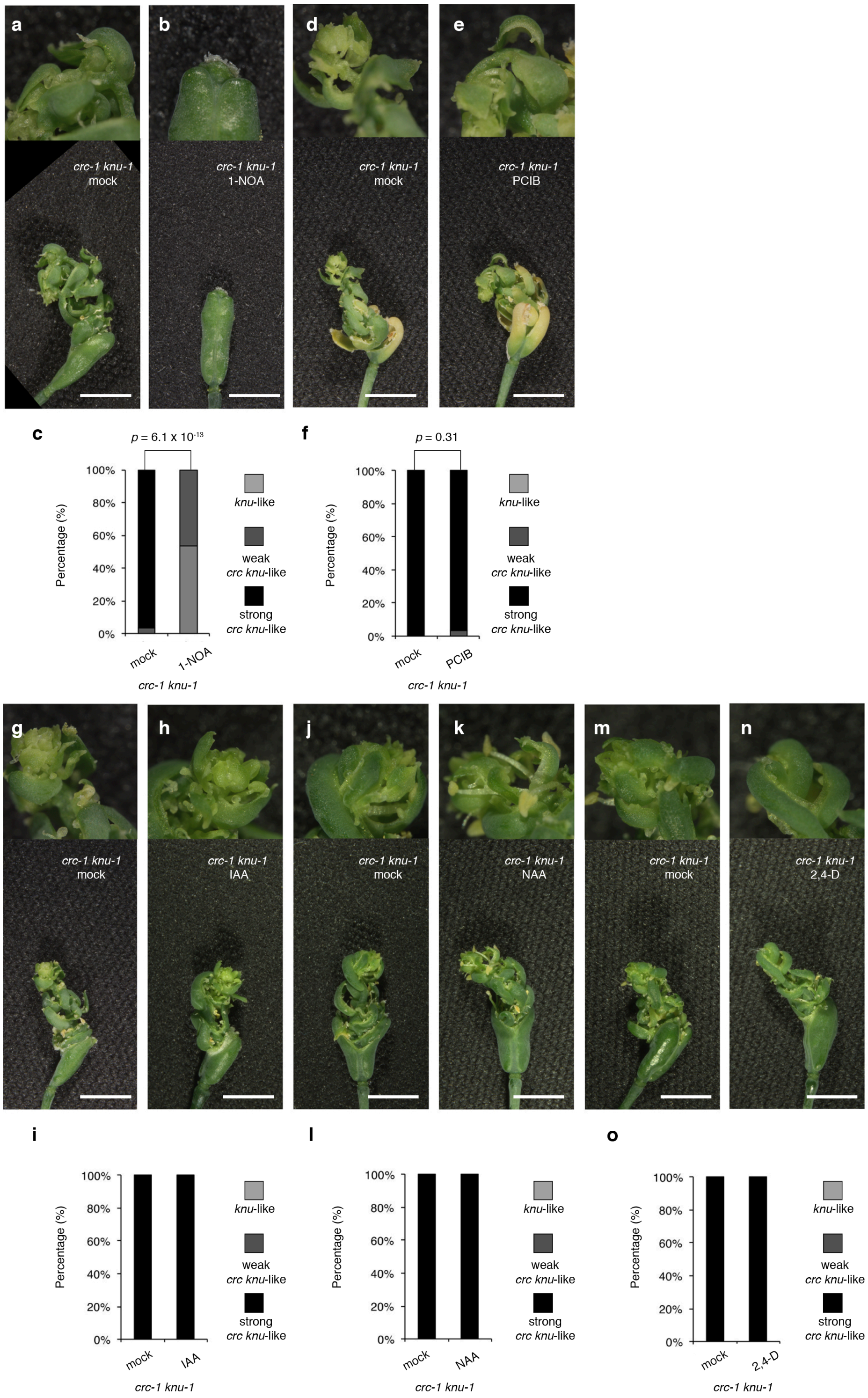

Supplementary Figure 7. *crc knu* mutant phenotypes after auxin and inhibitor treatments.

a-c, Morphology of mock-treated *crc-1 knu-1* (**a**) and 1-NOA-treated *crc-1 knu-1* (**b**) fruits. Quantification of mutant phenotypes after 1-NOA treatment (**c**). **d-f,** Morphology of mock-treated *crc-1 knu-1* (**d**), and PCIB-treated *crc-1 knu-1* (**e**) fruits. Quantification of mutant phenotypes after PCIB treatment (**f**). **g-i,** Morphology of mock-treated *crc-1 knu-1* (**g**), and IAA-treated *crc-1 knu-1* (**h**) fruits. Quantification of mutant phenotype after IAA treatment (**i**). **j-l,** Morphology of mock-treated *crc-1 knu-1* (**j**), and NAA-treated *crc-1 knu-1* (**k**) fruits. Quantification of mutant phenotype after NAA treatment (**l**). **m-o,** Morphology of mock-treated *crc-1 knu-1* (**m**), and 2,4-D-treated *crc-1 knu-1* (**n**) fruits. Quantification of mutant phenotypes after 2,4-D treatment (**o**). *p*-values were calculated by a Chi-square test (n = 30). Bars = 1 cm in **a**, **b, d, e, g, h, j, k, m, n** bottom.

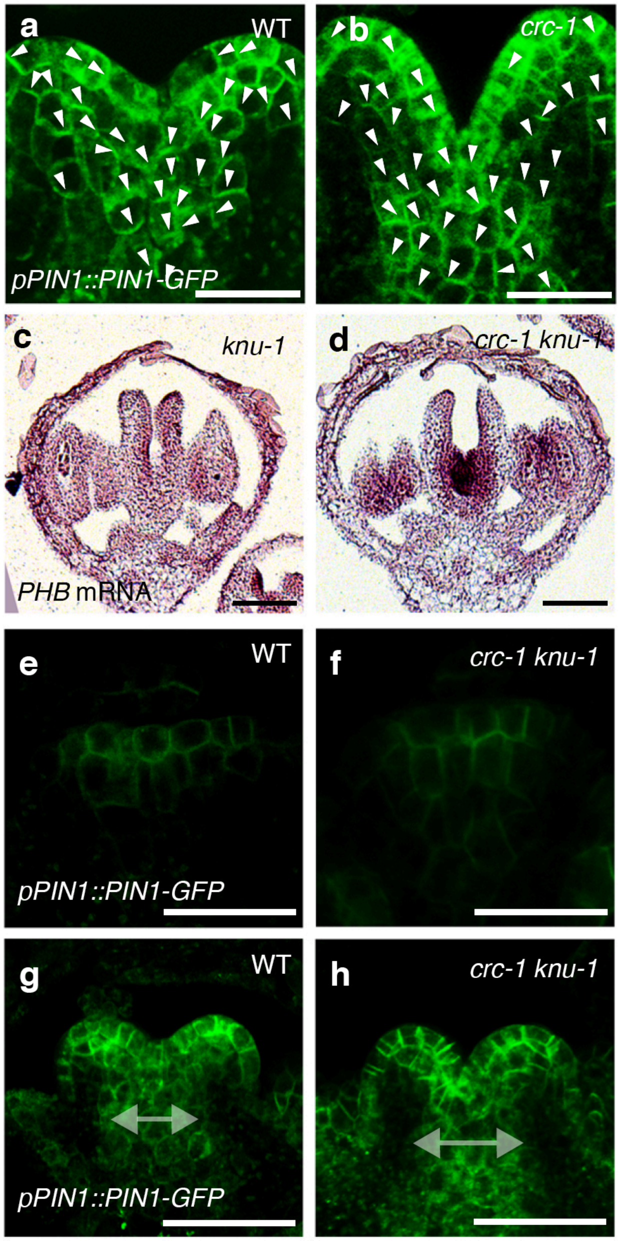

Supplementary Figure 8. PIN1-GFP, and *PHB* expression in mutants.

a, **b**, *pPIN1::PIN1-GFP* expression in wild-type (**a**) and *crc-1* (**b**) floral buds at stage 6. **c, d**, *In situ* hybridization showing the distribution of the adaxial fate marker *PHABULOSA* (*PHB*) in longitudinal sections of *knu-1* (**c**), and *crc-1 knu-1* (**d**) floral buds at developmental stages 9. **e-g**, *pPIN1::PIN1-GFP* expression in wild-type (**e, g**) and *crc-1 knu-1* (**f, h**) floral buds at stage 5 (**e, f**) and 6 (**g, h**). Prior to floral meristem termination, no obvious difference was observed. Bars = 10 µm in **a, b**; 100 µm in **c, d**; 25 µm in **e, f**; and 50 µm in **g, h.**

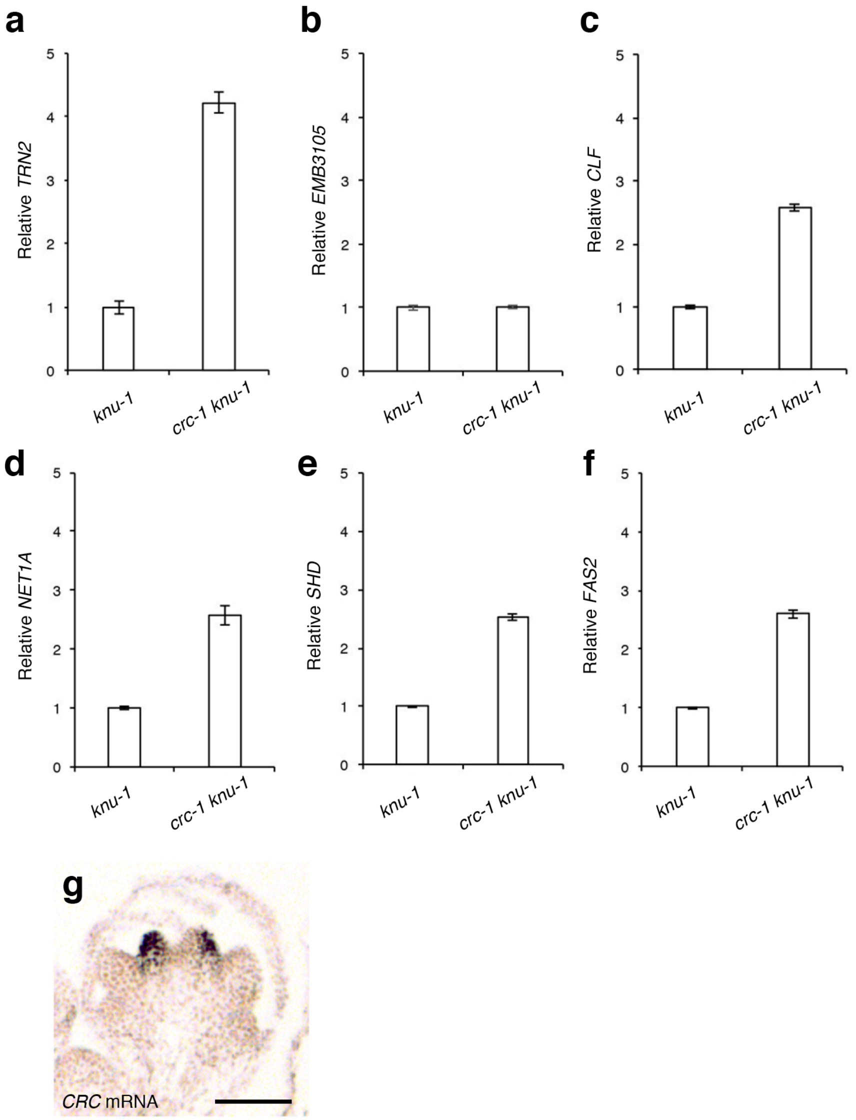

Supplementary Figure 9. Expression of the final six CRC target candidates.

a-**f**, *TRN2* (**a**), *EMB3105* (**b**), *CLF* (**c**), *NET1A* (**d**), *SHD* (**e**), and *FAS2* (**f**) mRNA abundance in *knu-1* and *crc-1 knu-1* flowers. The mean and standard error of the mean of each gene expression level were normalized to that of *EIF4* (*At3g13290*). **g**, *In situ* hybridization showing the distribution of *CRC* transcript in longitudinal sections of wild-type floral buds at developmental stages 6. Bars = 100 μm in **g**.

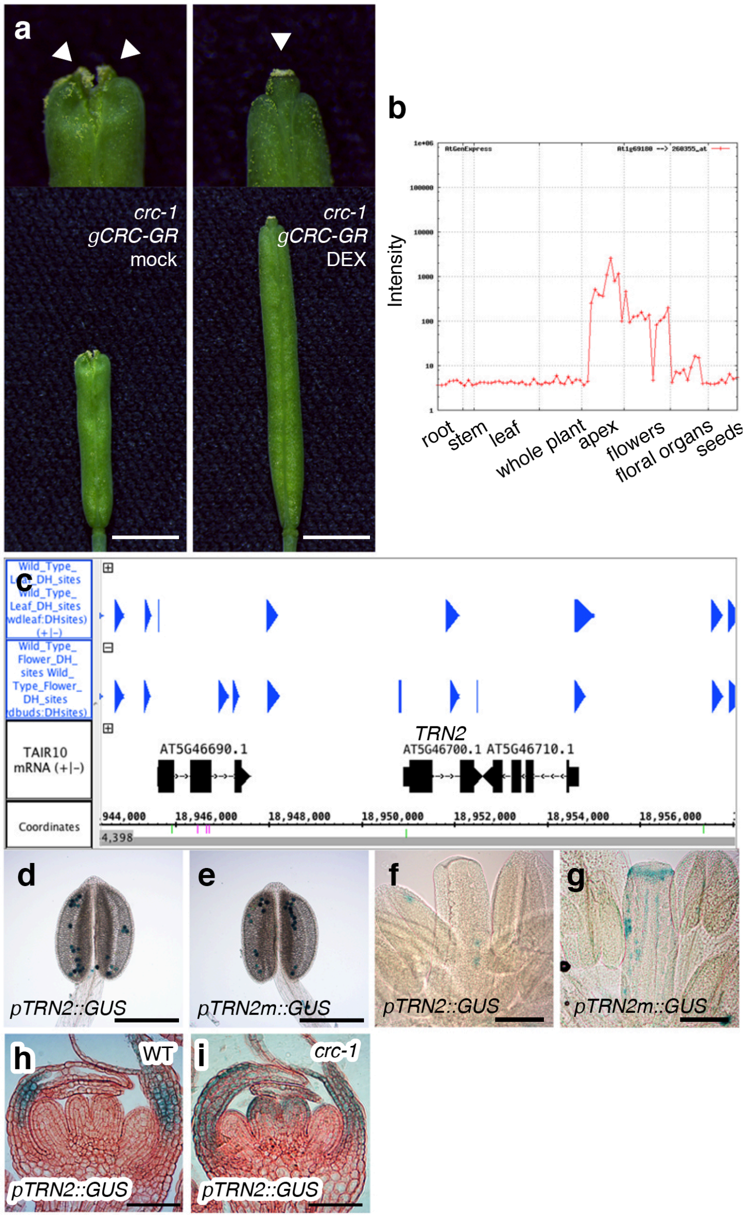

Supplementary Figure 10. The flower-specific transcription factor CRC control *TRN2* expression via the YABBY-binding site.

a, Phenotypic rescue of *crc-1* mutant by *gCRC-GR*. Morphology of mock- (left) and DEX- (right) treated *crc-1 gCRC-GR*. Arrowheads indicate stigma structures. **b**, *CRC* expression based on AtGenExpress. *CRC* is not expressed in leaves. *CRC* is highly expressed in inflorescences and flowers. For detailed sample descriptions, see http://www.weigelworld.org/resources/microarray/AtGenExpress/AtGE_dev_samples.pdf/view. **c**, YABBY-binding site identification by DNase I HS assay and sequences of potential YABBY-binding sites. Pink, GA[GA]AGAAA; Green,

CC[CA][TC]C[TA][CT]C; Not found, CCCCAC. GA[A/G]AGAAA sequence (Pink) was not observed elsewhere in the *TRN2* locus. Also, two other potential YABBY-binding sites (CC[C/A][T/C]C[T/A][C/T]C (Green) or CCCCAC) were not found anywhere in the *TRN2* locus. **d-g**, Reporter expression of *TRN2* from wild-type (**d**, **f**) and YABBY-binding site-mutated (**e, g**) *TRN2* promoters in inflorescences pollen (**d**, **e**) and flowers (**f**, **g**). GUS staining in *pTRN2::GUS* plants was observed in pollen, as reported previously. In *pTRN2m*::*GUS* plants, *GUS* expression was higher in older flowers and was detected as a continuous apical ring in stage 9 gynoecia and in longitudinal strands at the sites of developing vasculature, whereas very weak signals were detected in *pTRN2::GUS* plants. **h**, **i**, *pTRN2::GUS* expression in wild-type (**h**) and *crc-1* (**i**) floral buds. Bars = 1 cm in **a** bottom; 100 µm in **d-g**; and 100 µm in **h**, **i**.

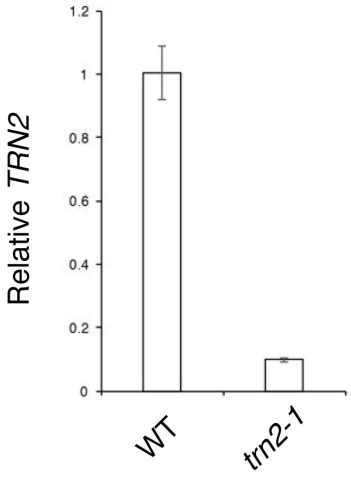

Supplementary Figure 11. Evaluation of *TRN2* expression by RT-PCR.

TRN2 mRNA abundance in wild-type and *trn2-1* flowers. The mean and standard error of the mean of *TRN2* expression were normalized to that of *EIF4* (*At3g13290*). The reduced *TRN2* expression in loss-of-function *trn2-1* suggests the presence of nonsense-mediated RNA decay.

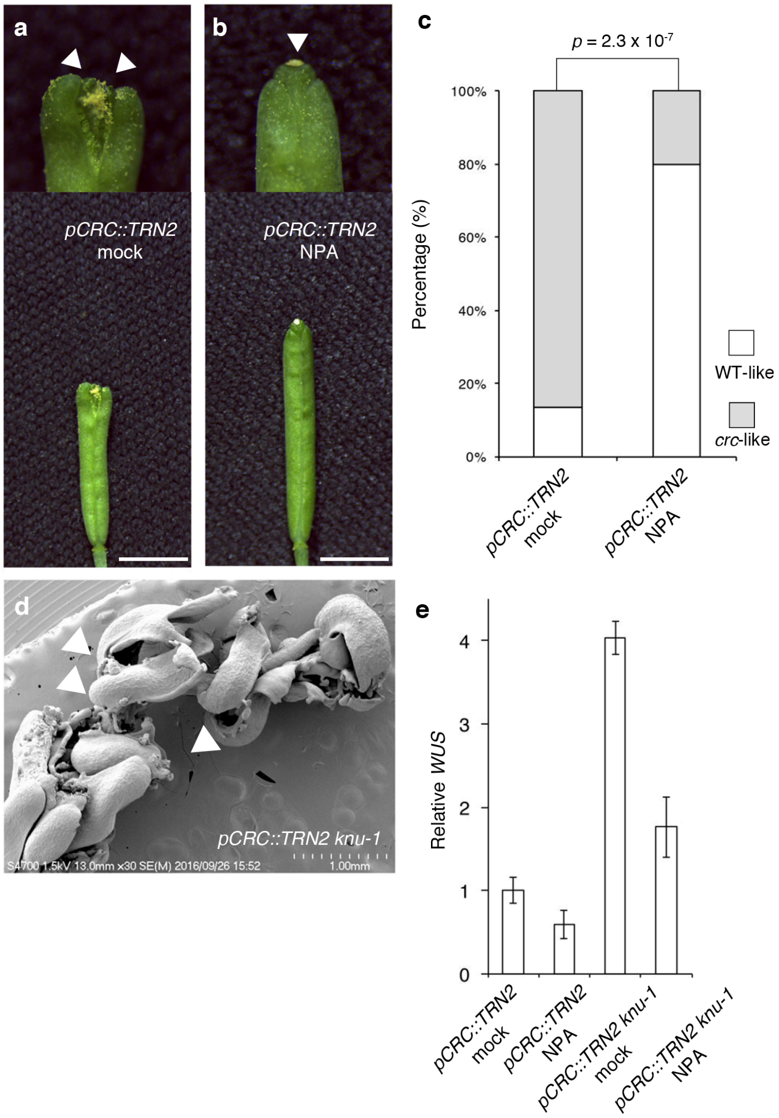

Supplementary Figure 12. Quantification of *pCRC::TRN2 knu-1* phenotypes after NPA treatment.

a, Morphology of mock-treated *pCRC::TRN2*. Arrowheads indicate stigma structures. **b**, NPA-treated *pCRC::TRN2*. **c**, Quantification of mutant phenotypes. **d,** Scanning electron micrograph of a *pCRC::TRN2 knu-1* fruit with reiterations of floral organs. Arrowheads indicate organ formation at different places on the internode. **e**, mRNA abundance of the stem cell marker *WUS* in mock-treated *pCRC::TRN2*, NPA-treated *pCRC::TRN2*, mock-treated *pCRC::TRN2 knu-1*, and NPA-treated *pCRC::TRN2 knu-1* flowers. NPA-treated *pCRC::TRN2* plants had slightly reduced levels of *WUS* mRNA relative to control plants. NPA treatment of *pCRC::TRN2 knu* plants also led to significantly reduced *WUS* mRNA levels compared to mock-treated *pCRC::TRN2 knu-1* plants. Bars = 1 cm in **a**, **b** bottom.

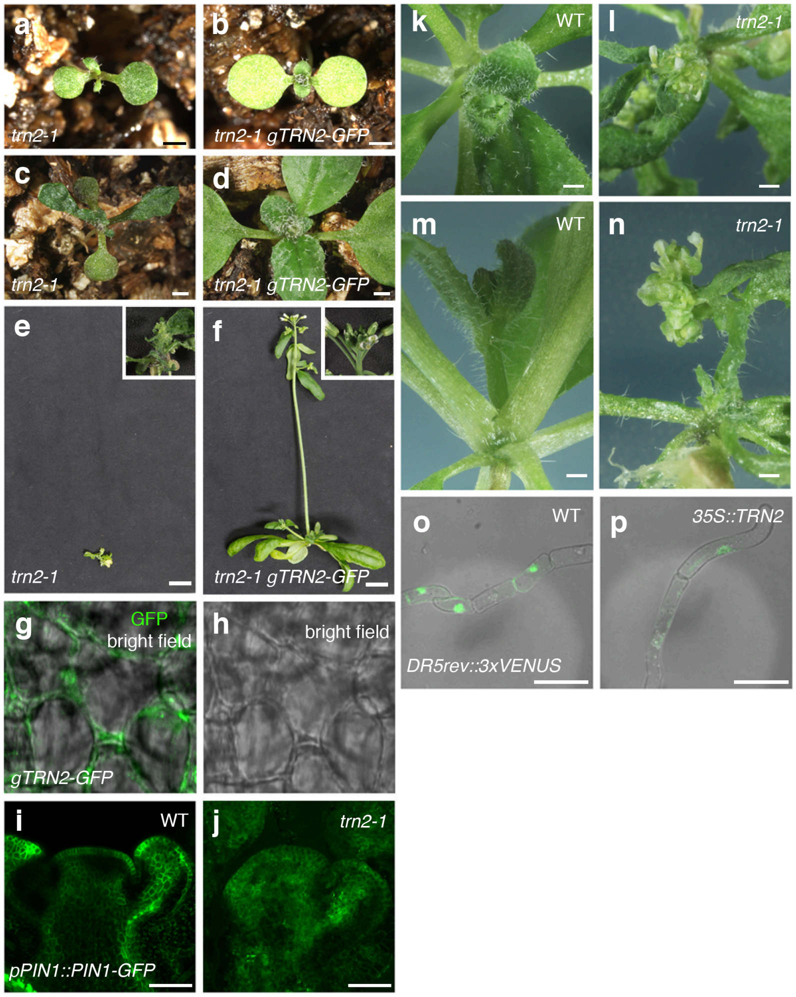

Supplementary Figure 13. The role of TRN2 in the auxin pathway.

a–f, Phenotypic rescue of *trn2-1* by *gTRN2-GFP*. **a, b,** Ten-day-old plants of *trn2-1* (**a**) and *trn2-1 gTRN2-GFP* (**b**). **c, d,** Twenty-day-old plants of *trn2-1* (**c**) and *trn2-1 gTRN2-GFP* (**d**). **e, f,** Thirty-five-day-old plants of *trn2-1* (**e**) and *trn2-1 gTRN2-GFP* (**f**). **g**, **h**, Merged image of *gTRN2-GFP* expression and bright field (**g**) and bright field image (**h**) in cells of wild-type floral primordia at stage 2. **i**, **j**, *pPIN1::PIN1-GFP* expression in wild-type (**i**) and *trn2-1* (**j**) inflorescences. **k–n**, Wild-type (**k, m**) and *trn2-1* (**l, n**) inflorescences grown on MS plates. (**k, l**) Top views. (**m, n**) Side views. **o**, **p**, *DR5rev::3xVENUS* expression in wild-type (**o**) and *35S::TRN2*-expressing (**p**) tobacco BY-2 cells. Bars = 1 mm in **a–d** and **k–n**; 1 cm in **e**, **f**; 50 µm in **i, j**; and 25 µm in **o**, **p**.

AGI code	Full name	Abbreviation	description	GO terms
AT1G48350	*EMBRYO DEFECTIVE 3105*	*EMB3105*	Ribosomal L18p/L5e family protein	GO:0009941 chloroplast envelope<br>GO:0009832 plant-type cell wall biogenesis<br>GO:0005840 ribosome<br>GO:0009664 plant-type cell wall organization<br>GO:0009570 chloroplast stroma<br>GO:0010075 regulation of meristem growth<br>GO:0042254 ribosome biogenesis<br>GO:0003735 structural constituent of ribosome<br>GO:0007389 pattern specification process<br>GO:0008361 regulation of cell size<br>GO:0009926 auxin polar transport<br>GO:0010015 root morphogenesis<br>GO:0048653 anther development<br>GO:0006412 translation
AT2G23380	*CURLY LEAF*	*CLF*	Similar to the product of the Polycomb-group gene	GO:0006306 DNA methylation<br>GO:0006349 regulation of gene expression by genetic imprinting<br>GO:0045857 negative regulation of molecular function, epigenetic<br>GO:0003700 transcription factor activity<br>GO:0005634 nucleus<br>GO:0009965 leaf morphogenesis<br>GO:0003727 single-stranded RNA binding<br>GO:0010048 vernalization response<br>GO:0031047 gene silencing by RNA<br>GO:0005515 protein binding<br>GO:0010228 vegetative to reproductive phase transition of meristem<br>GO:0051567 histone H3-K9 methylation<br>GO:0009294 DNA mediated transformation
AT4G24190	*SHEPHERD*	*SHD*	encodes an ortholog of	GO:0006457 protein folding<br>GO:0005783 endoplasmic reticulum

			GRP94, an ER-resident HSP90-like protein	GO:0048046 apoplast<br>GO:0005739 mitochondrion<br>GO:0009414 response to water deprivation<br>GO:0009651 response to salt stress<br>GO:0006096 glycolysis<br>GO:0006094 gluconeogenesis<br>GO:0005634 nucleus<br>GO:0051082 unfolded protein binding<br>GO:0005774 vacuolar membrane<br>GO:0046686 response to cadmium ion<br>GO:0005524 ATP binding<br>GO:0009306 protein secretion<br>GO:0006833 water transport<br>GO:0006972 hyperosmotic response<br>GO:0009409 response to cold<br>GO:0009408 response to heat<br>GO:0009644 response to high light intensity<br>GO:0009506 plasmodesma<br>GO:0009507 chloroplast<br>GO:0007030 Golgi organization<br>GO:0010075 regulation of meristem growth<br>GO:0042542 response to hydrogen peroxide<br>GO:0006499 N-terminal protein myristoylation<br>GO:0009934 regulation of meristem structural organization<br>GO:0009934 regulation of meristem structural organization
AT3G22790	*NETWORKED 1A*	*NET1A*	Encodes a member of the NET superfamily of proteins .	GO:0042127 regulation of cell proliferation<br>GO:0000911 cytokinesis by cell plate formation<br>GO:0010089 xylem development<br>GO:0010073 meristem maintenance<br>GO:0009965 leaf morphogenesis

				GO:0051015 actin filament binding<br>GO:0044036 cell wall macromolecule metabolic process<br>GO:0000226 microtubule cytoskeleton organization<br>GO:0005884 actin filament<br>GO:0009855 determination of bilateral symmetry<br>GO:0010014 meristem initiation<br>GO:0009506 plasmodesma
AT5G46700	*TORNADO 2*	*TRN2*	Encodes a transmembrane protein of the tetraspanin (TET) family	GO:0009956 radial pattern formation<br>GO:0009944 polarity specification of adaxial/abaxial axis<br>GO:0007568 aging<br>GO:0010075 regulation of meristem growth<br>GO:0010305 leaf vascular tissue pattern formation<br>GO:0016021 integral to membrane<br>GO:0009855 determination of bilateral symmetry<br>GO:0008361 regulation of cell size<br>GO:0019344 cysteine biosynthetic process<br>GO:0010014 meristem initiation<br>GO:0010015 root morphogenesis<br>GO:0009926 auxin polar transport<br>GO:0009506 plasmodesma
AT5G64630	*FASCIATA2*	*FAS2*	Chromatin Assembly Factor-1 p60 subunit	GO:0031507 heterochromatin formation<br>GO:0009555 pollen development<br>GO:0000724 double-strand break repair via homologous recombination<br>GO:0006334 nucleosome assembly<br>GO:0008283 cell proliferation<br>GO:0000166 nucleotide binding<br>GO:0009933 meristem structural organization<br>GO:0005515 protein binding<br>GO:0033186 CAF-1 complex

				GO:0010026 trichome differentiation

Supplementary Table 1. GO term list of six selected CRC downstream candidate genes.

Primer name	Sequence	Notes
Genotyping		
trn2-1-FW	CGTCCCAACTTTCTAGATTCTTC	EcoRV
trn2-1-RV	CATATCAGCAGACATATCTATGGGAgaTAT	
crc-1-FW	CCTTTGACATATACTCTTTAGTTCC	HindIII
crc-1-RV	CTTTTGATGCGTTGGATCTCAagC	
knu-1-FW	CCCGCCGTCTCTTTCCATGTCAGTACT	HpyCH4III
knu-1-RV	CGACGAGCAGCGGCGCGTTCGC	
wus-1-FW	TTGAATTAATGAATTATAGTTTGATACG	NcoI
wus-1-RV	TTGAAGTTATGGATCTTGATTGG	
RT-PCR		
CRC-FW	TCCCTTTGTCGTCAAACCTC	
CRC-RV	CCCTCATGAAGCGGTTGTAT	
EIF4-FW	ACCAGGCGTAAGGTTGATTG	
EIF4-RV	GGTCCATGTCTCCGTGAGTT	
WUS-FW	GCAAGCTCAGGTACTGAATGTGGTG	
WUS-RV	GACCAAACAGAGGCTTTGCTCTATCG	
TRN2-FW	ATCCGTGAAGAGTACCCAGAT	
TRN2-RV	AAGAACCATGCACTCATCAGC	
EMB3105-FW	AGTCTCGGTCCATGGTTGTC	
EMB3105-RV	ATTCTGTCTTCGCTGCTGGT	
CLF-FW	ACCACACCCACGAAGTTCTC	
CLF-RV	TGCGCACTTTTCATTAGACG	
NET1A-FW	GAGAAGCCAGGCAAGTTCAC	
NET1A-RV	GCAAGCTGAAAGATGGCTTC	
SHD-FW	CAAGAGCGGTCTGAACATCA	
SHD-RV	TGGTCTCTGGTTCCTCTGCT	
FAS2-FW	GTTTTTGCCCTGTTGCATTT	
FAS2-RV	TGCAATGGCAAAGACAAGAC	

ChIP		
CRC-I-FW	CGTCCTGAGGCAAGTGAAAG	
CRC-I-RV	CGTCATCATTGCATCGCATAG	
CRC-II-FW	CGTATTGGGACTTAACTCACAAAG	
CRC-III-FW	GTGGGTTTGGTGAACTTTCC	
CRC-III-RV	CTAAATAAGTGGAGGTGCAACATC	
CRC-IV-FW	GGCAACGCTTTAAAGAGTCG	
CRC-IV-RV	GGGGACTATCAACTATGACTG	
CRC-V-FW	CCATCTCTCACAGTCATAGTTG	
CRC-V-RV	GTACGTAATAGGGTAATCGTGG	
CRC-VI-FW	CGTCTCATTATTTCTAGTCCACG	
CRC-VI-RV	CTCATCATTGGCATTAAGAGACA	
CRC-VII-FW	CTTGAGGATAATTTCTCATTGTCC	
CRC-VII-RV	CCACTGTACGGTAGTCTAATTCC	
CRC-VIII-FW	CAATAAAGCGTGTATAGCTCCC	
CRC-VIII-RV	CTGTTTGAGGTTATGAGGGTTAAC	
MU-FW	GATTTACAAGGAATCTGTTGGTGGT	
TRN2-I-FW	TAAGACATCCGCCATCATTG	
TRN2-I-RV	AGTGTCGTGGATGTTGATGG	
TRN2-II-FW	CGCGTAATCTCTCCCGTTTA	
TRN2-II-RV	AGTACCTCCAAATCGGCAAG	
TRN2-III-FW	ACTTCATCACCGTCCTCCTC	
TRN2-III-RV	ACTGGCCATTGAAGAAGCTTG	
Cloning		
gCRC-FW	GGggtaccCGCTCGTCTCCTCATAACCG	
gCRC-RV	CCgggcccCTTCTTCTCACCGAATCCCAAG	
CRC CDS-FW	CGACGCGTCGCTAAAGACCATGAACCTAG	
CRC CDS-RV	CCgggcccCTTCTTCTCACCGAATCCCAAG	
pCRC-FW	GGggtaccGTCAAATCAGAAAGAGAGCG	

pCRC-RV	CCGCTCGAGGGTCTTTAGCGAATGGATTG	
gTRN2-FW	GGCGCGCCTCAGTAACTAGGGTGATACAAAC	
gTRN2-RV	ccGGGCCCAGTATAACCCTGCTTGTACTTC	
TRN2 3'UTR-FW	gcTCTAGATCAAGATGAGAAGTTTAGTGGC	
TRN2 3'UTR-RV	gcTCTAGAGCGGCCGCGTCAGATGGTCAAATTCGAGTAC	
TRN2 CDS-FW	ccgCTCGAGATGCCTTTAAGCAACAATGTAATTG	
TRN2 CDS-RV	ccGGGCCCAGTATAACCCTGCTTGTACTTC	
pTRN2-FW	caccATAGTAATTAAGTTATAAATTAG	
pTRN2-RV	CTTTTTTGGGAGAGATGAGAGTTTTAAC	
pTRN2m-FW	CTAggatccTCTCTCTATTTCAAACCAAAC	
pTRN2m-RV	TAGAGAGAggatccTAGAATTGAACTTAAAG	
iaaH-FW	ACGCGTCGACATGGTGGCCATTACCTCGTT	
iaaH-RV	ccGGGCCCATTGGGTAAACCGGCAAAATATC	

Supplementary Table 2. Primers used in this study.
