# Supplementary material for: Fine-tuning of auxin homeostasis governs the transition from floral stem cell maintenance to gynoecium formation
Source: Nat Commun. 2017 Oct 24;8:1125. doi: 10.1038/s41467-017-01252-6 (PMC5654772; doi:10.1038/s41467-017-01252-6)
Supplement: Supplementary file 3 — Description of Additional Supplementary Files [file 41467_2017_1252_MOESM3_ESM.pdf]

Description of Supplementary Files

File Name: Supplementary Data 1
Description: High confident upregulated and downregulated genes in *crc knu*.

File Name: Supplementary Data 2
Description: Go term analysis of upregulated genes identified by agriGO.
